# Acute and postacute sequelae associated with SARS-CoV-2 reinfection

**DOI:** 10.1038/s41591-022-02051-3

**Published:** 2022-11-10

**Authors:** Benjamin Bowe, Yan Xie, Ziyad Al-Aly

**Affiliations:** 1 Clinical Epidemiology Center, Research and Development Service, Veteran Affairs Saint Louis Health Care System, St. Louis, MO USA; 2 Veterans Research and Education Foundation of St. Louis, St. Louis, MO USA; 3 Department of Medicine, Washington University School of Medicine, St. Louis, MO USA; 4 Nephrology Section, Medicine Service, Veteran Affairs St. Louis Health Care System, St. Louis, MO USA; 5 Institute for Public Health, Washington University in St. Louis, St. Louis, MO USA

**Keywords:** SARS-CoV-2, Viral infection

## Abstract

First infection with severe acute respiratory syndrome coronavirus 2 (SARS-CoV-2) is associated with increased risk of acute and postacute death and sequelae in various organ systems. Whether reinfection adds to risks incurred after first infection is unclear. Here we used the US Department of Veterans Affairs’ national healthcare database to build a cohort of individuals with one SARS-CoV-2 infection (*n* = 443,588), reinfection (two or more infections, *n* = 40,947) and a noninfected control (*n* = 5,334,729). We used inverse probability-weighted survival models to estimate risks and 6-month burdens of death, hospitalization and incident sequelae. Compared to no reinfection, reinfection contributed additional risks of death (hazard ratio (HR) = 2.17, 95% confidence intervals (CI) 1.93–2.45), hospitalization (HR = 3.32, 95% CI 3.13–3.51) and sequelae including pulmonary, cardiovascular, hematological, diabetes, gastrointestinal, kidney, mental health, musculoskeletal and neurological disorders. The risks were evident regardless of vaccination status. The risks were most pronounced in the acute phase but persisted in the postacute phase at 6 months. Compared to noninfected controls, cumulative risks and burdens of repeat infection increased according to the number of infections. Limitations included a cohort of mostly white males. The evidence shows that reinfection further increases risks of death, hospitalization and sequelae in multiple organ systems in the acute and postacute phase. Reducing overall burden of death and disease due to SARS-CoV-2 will require strategies for reinfection prevention.

## Abstract (web summary)

A new analysis using US Department of Veterans Affairs databases showed that reinfection is associated with increased risk of all-cause mortality, hospitalization and a wide range of long COVID complications in individuals who have had SARS-CoV-2 compared to those with no reinfection.

## Main

A large body of evidence suggests that first infection with SARS-CoV-2 is associated with increased risk of acute and postacute death and sequelae in the pulmonary and broad array of extrapulmonary organ systems^1–8^. However, many people around the globe are experiencing repeat SARS-CoV-2 infections (reinfections). Previous epidemiological studies of SARS-CoV-2 reinfection have been limited to investigations of the risk of getting reinfection and the comparative evaluation of risk differences of hospitalization or death between first and second SARS-CoV-2 infections during their acute phase^9,10^. Whether and to what extent reinfection adds to the risk incurred after the first infection is not clear (that is, evaluation of the risk of reinfection versus no reinfection). Whether reinfection contributes to the increased risk of acute and postacute sequelae is also not known. Addressing these questions has broad public health implications since it will inform whether strategies to prevent or reduce the risk of reinfection should be implemented.

In this study, we used the electronic healthcare database of the US Department of Veterans Affairs to address the question of whether SARS-CoV-2 reinfection adds to the health risks associated with a first SARS-CoV-2 infection. We characterized the risks and 6-month burdens of a range of prespecified outcomes in a cohort of people who experienced a SARS-CoV-2 reinfection compared to those with no reinfection, characterized the risks of acute and postacute outcomes in people who had reinfection and finally estimated the cumulative risks and one-year burdens associated with one, two, three or more infections compared to a noninfected control cohort.

## Results

**Extended Data Fig. 1:**
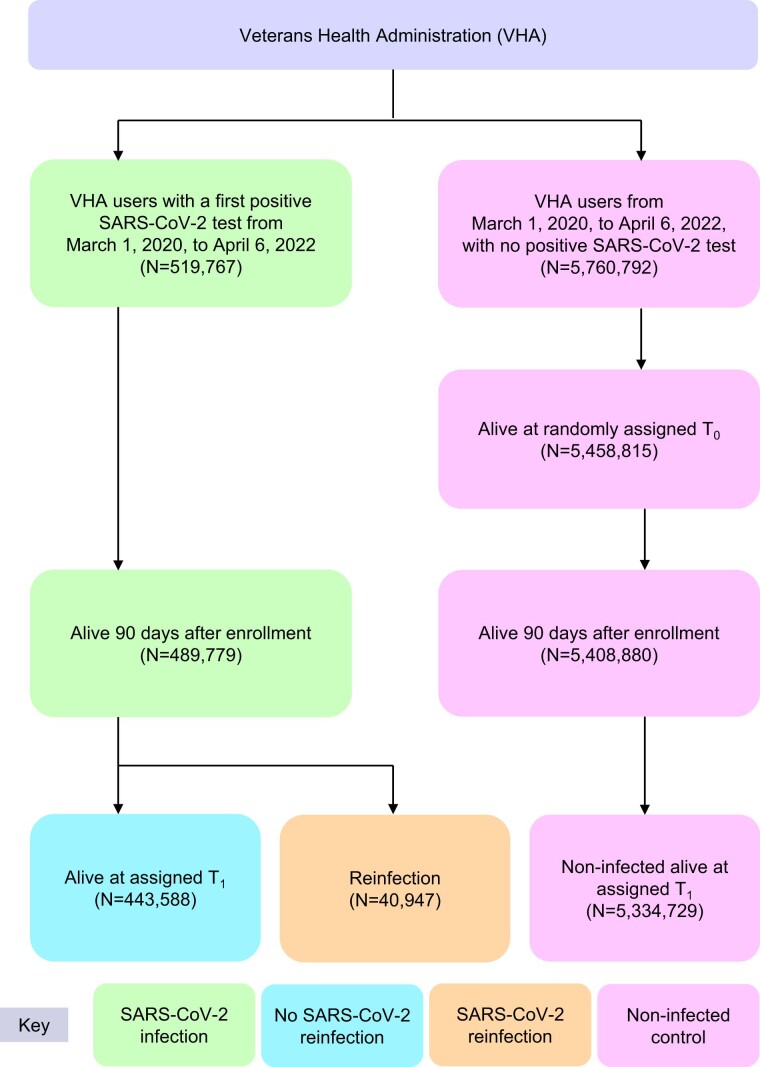
Cohort flowchart.

There were 443,588 cohort participants with no SARS-CoV-2 reinfection (only a single SARS-CoV-2 infection) and 40,947 participants who had SARS-CoV-2 reinfection (two or more infections) (Extended Data Fig. 1); 5,334,729 participants with no record of positive SARS-CoV-2 infection were in the noninfected control group. Among those who had reinfection, 37,997 (92.8%) people had two infections, 2,572 (6.3%) people had three infections and 378 (0.9%) people had four or more infections. The median distribution of time between the first and second infection was 191 d (interquartile range (IQR) = 127–330) and between the second and third was 158 d (IQR = 115–228). The demographic and health characteristics of those with no reinfection, reinfection and the noninfected control group are presented in Supplementary Table 1.

### Sequelae of SARS-CoV-2 reinfection

To gain a better understanding of whether reinfection adds risk, we first conducted analyses to examine the risks of all-cause mortality, hospitalization and a set of prespecified outcomes in people who had reinfection compared to those with no reinfection.

We provide two measures of risk: (1) we estimated the adjusted HRs of a set of incident prespecified outcomes comparing people who had reinfection versus no reinfection and (2) estimated the adjusted excess burden of each outcome per 1,000 persons 6 months after SARS-CoV-2 reinfection on the basis of the difference between the estimated incidence rate in individuals who had reinfection and no reinfection. Follow-up began at the time of reinfection, where reinfection was defined as a SARS-CoV-2 positive test at least 90 d after the initial positive test; this time frame of 90 d was specified to reduce the probability that a positive test was related to the first infection. Assessment of standardized mean differences of participant characteristics (from data domains including diagnoses, medications and laboratory test results) after application of weighting showed they were well balanced in each analysis of incident outcomes (Supplementary Table 2 and Supplementary Fig. 1).

**Fig. 1:**
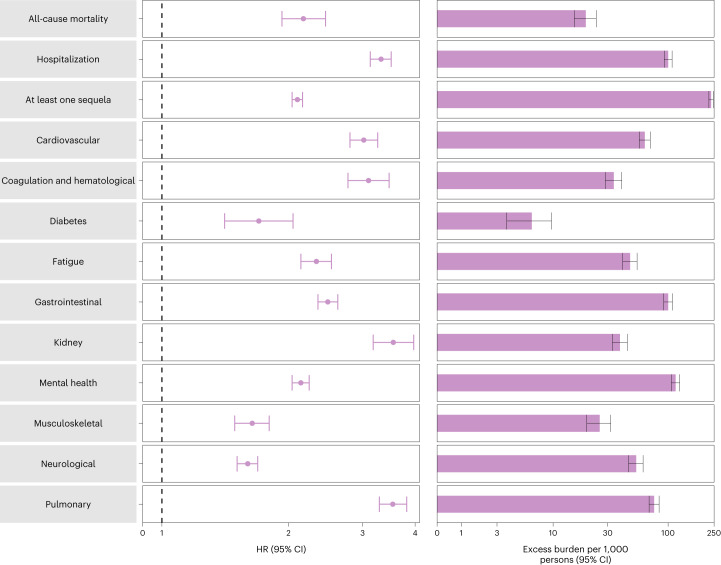
Risk and burden of sequelae in people with SARS-CoV-2 reinfection versus no reinfection. Risk and 6-month excess burden of all-cause mortality, hospitalization, at least one sequela and sequelae by organ system are plotted. Incident outcomes were assessed from reinfection to the end of the follow-up. Results from SARS-CoV-2 reinfection (*n* = 40,947) and no SARS-CoV-2 reinfection (*n* = 443,588) are compared. Adjusted HRs (dots) and 95% CIs (error bars) are presented, as are the estimated excess burden (bars) and 95% CIs (error bars). Burdens are presented per 1,000 persons at 6 months of follow-up from the time of reinfection.

Compared to those with no reinfection, those who had reinfection exhibited an increased risk of all-cause mortality (HR = 2.17, 95% CI = 1.93–2.45) and excess burden of all-cause mortality estimated at 19.33 (95% CI = 15.34–23.82) per 1,000 persons at 6 months; all burden estimates represent excess burden and are given per 1,000 persons at 6 months (Fig. 1 and Supplementary Table 3). People with a reinfection also had an increased risk of hospitalization (HR = 3.32, 95% CI = 3.13–3.51; a burden of 100.19 (92.53–108.25)) and having at least one sequela of SARS-CoV-2 infection (HR = 2.10, 95% CI = 2.04–2.16; a burden of 235.91 (225.54–246.34)) (Fig. 1 and Supplementary Table 3).

Compared to those with no reinfection, those who had reinfection exhibited increased risk of sequelae in the pulmonary (HR = 3.54, 95% CI = 3.29–3.82; burden = 75.74, 95% CI = 68.47–83.50) and several extrapulmonary organ systems including cardiovascular disorders (HR = 3.02, 95% CI = 2.80–3.26; burden = 62.80, 95% CI = 56.17–69.91), coagulation and hematological disorders (HR = 3.10, 95% CI = 2.77–3.47; burden = 33.85, 95% CI = 28.55–39.74), fatigue (HR = 2.33, 95% CI = 2.14–2.53; burden = 46.92, 95% CI = 40.46–53.89), gastrointestinal disorders (HR = 2.48, 95% CI = 2.35–2.62; burden = 100.30, 95% CI = 91.88–109.09), kidney disorders (HR = 3.55, 95% CI = 3.18–3.97; burden = 38.31, 95% CI = 32.86–44.37), mental health disorders (HR = 2.14, 95% CI = 2.04–2.24; burden = 116.13, 95% CI = 106.71–125.87), diabetes (HR = 1.70, 95% CI = 1.41–2.05; burden = 6.46, 95% CI = 3.77–9.69), musculoskeletal disorders (HR = 1.64, 95% CI = 1.49–1.80; burden = 25.55, 95% CI = 19.73–31.91) and neurological disorders (HR = 1.60, 95% CI = 1.51–1.69; burden = 52.91, 95% CI = 45.48–60.70). Risks and excess burdens of reinfection are provided in Fig. 1 and Supplementary Table 3. Analyses examining whether the length of time from first infection to reinfection might modify the association between reinfection and the risks of all-cause mortality, hospitalization and at least one sequela suggested no effect modification on the multiplicative scale (*P* values for effect modification of 0.224, 0.156 and 0.356, respectively).

**Fig. 2:**
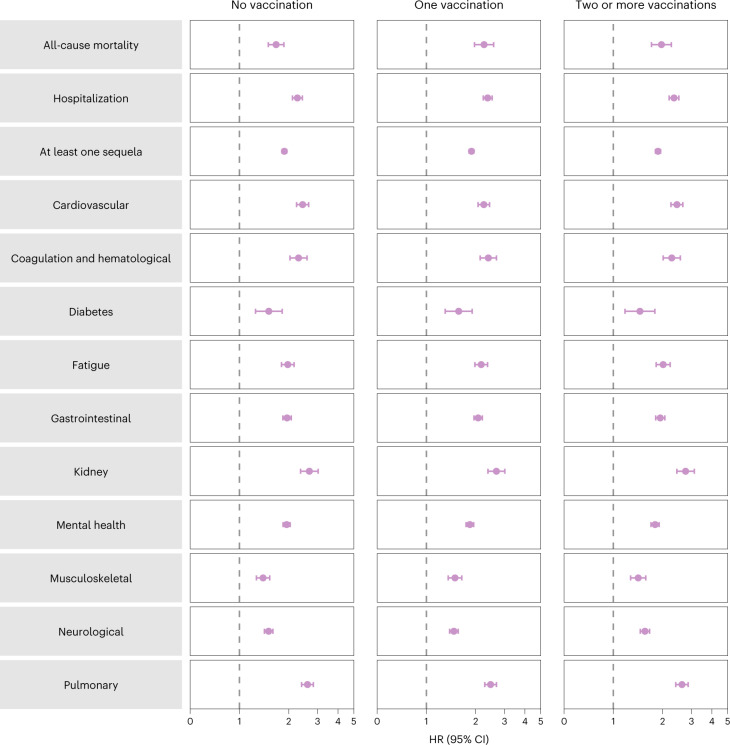
Risk and burden of sequelae in people with SARS-CoV-2 reinfection versus no reinfection by vaccination status before reinfection. Risk of all-cause mortality, hospitalization, at least one sequela and sequelae by organ system are plotted. Incident outcomes were assessed from reinfection to the end of the follow-up. Results from SARS-CoV-2 reinfection (*n* = 40,947) versus no SARS-CoV-2 reinfection (*n* = 443,588) are compared. At the time of comparison, there were 51.3%, 12.6% and 36.2% with no, one and two or more vaccinations, respectively, among those who had reinfection. At the time of comparison, there were 41.1%, 11.7% and 47.2% with no, one and two or more vaccinations, respectively, among the no reinfection group. Adjusted HRs (dots) and 95% CIs (error bars) are presented.

Analyses of prespecified subgroups based on vaccination status before reinfection (no vaccination, one vaccination or two or more vaccinations) showed that reinfection (compared to no reinfection) was associated with a higher risk of all-cause mortality, hospitalization, at least one sequela and sequelae in the different organ systems (Fig. 2 and Supplementary Table 4) regardless of vaccination status.

### Acute and postacute sequelae of SARS-CoV-2 reinfection

**Fig. 3:**
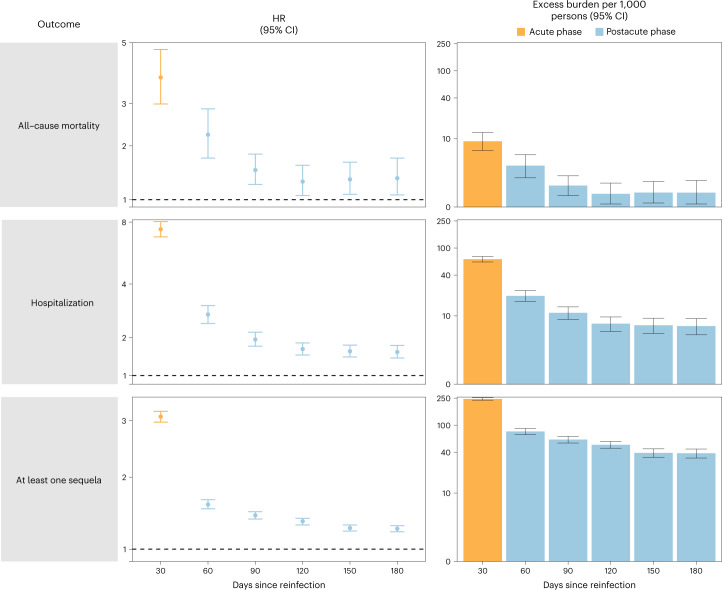
Risk and burden of all-cause mortality, hospitalization and at least one sequela in the acute and postacute phases of SARS-CoV-2 reinfection versus no reinfection. Risk and 6-month burden of all-cause mortality, hospitalization and at least one sequela of SARS-CoV-2 reinfection versus no reinfection in 30-d intervals covering the acute and postacute phases of reinfection. Incident outcomes were assessed from reinfection to the end of the follow-up. Results from SARS-CoV-2 reinfection (*n* = 40,947) versus first SARS-CoV-2 infection (*n* = 443,588) by time since reinfection were compared. Adjusted HRs (dots) and 95% CIs (error bars) are presented for each 30-d period since the time of reinfection, as are the estimated excess burden (bars) and 95% CIs (error bars). Burdens are presented per 1,000 persons at every 30-d period of the follow-up from the time of reinfection.

**Fig. 4:**
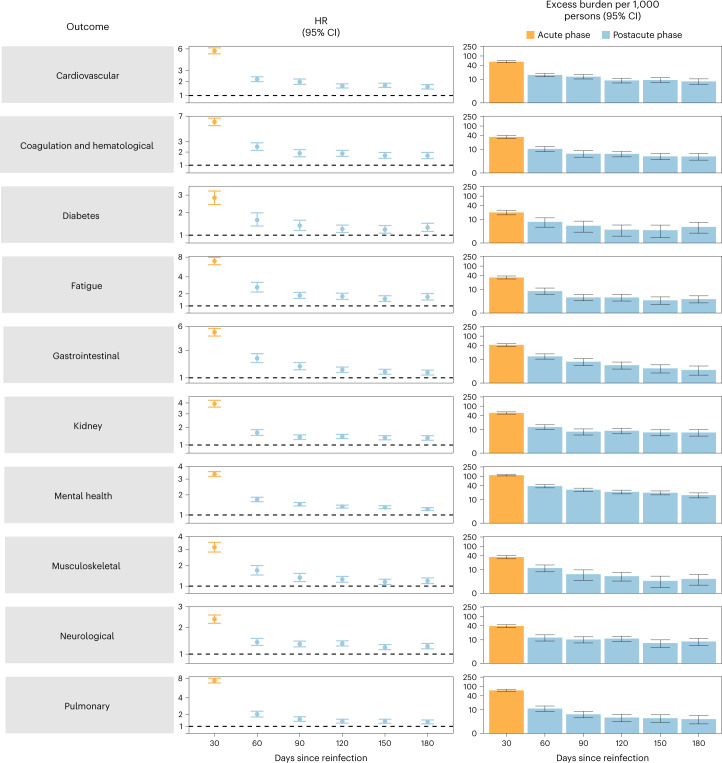
Risk and burden of sequelae by organ system in the acute and postacute phases of SARS-CoV-2 reinfection versus no reinfection. Risk and 6-month excess burden of sequelae by organ system of SARS-CoV-2 reinfection versus no reinfection in 30-d intervals covering the acute and postacute phases of reinfection. Incident outcomes were assessed from reinfection to the end of the follow-up. Results from SARS-CoV-2 reinfection (*n* = 40,947) versus first SARS-CoV-2 infection (*n* = 443,588) by time since reinfection are compared. Adjusted HRs (dots) and 95% CIs (error bars) are presented for each 30-d period since the time of reinfection, as are the estimated excess burden (bars) and 95% CIs (error bars). Burdens are presented per 1,000 persons at every 30-d period of the follow-up from the time of reinfection.

We examined whether the risk of sequelae of SARS-CoV-2 reinfection was present in the acute and postacute phases of reinfection. We conducted analyses examining risk and burden starting from the time of reinfection up to 180 d later in 30-day increments. Compared to those with no reinfection, those who had reinfection exhibited increased risk and excess burden of all-cause mortality, hospitalization and at least one sequela in the acute and postacute phases of reinfection. The risks and excess burdens of all-cause mortality, hospitalization and at least one sequela during the postacute phase gradually attenuated over time but remained evident even 6 months after reinfection (Fig. 3 and Supplementary Table 5). Examination of sequelae by organ system suggested an increased risk and excess burden in all organ systems during the acute phase (Fig. 4 and Supplementary Table 5). The risks and burdens persisted in the postacute phase of reinfection and were still evident at 6 months after reinfection.

### Cumulative risk and burden of one, two and three or more SARS-CoV-2 infections

**Fig. 5:**
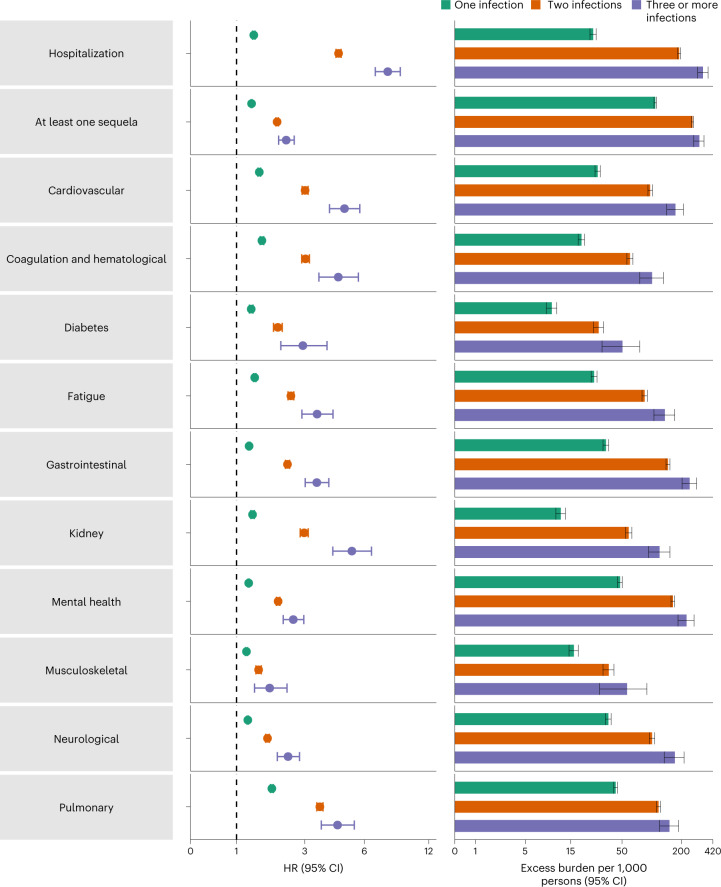
Cumulative risk and burden of sequelae in people with one, two and three or more SARS-CoV-2 infections compared to noninfected controls. Risk and 1-year excess burden of hospitalization, at least one sequela and sequelae by organ system are plotted. Incident outcomes were assessed from 30 d after the first positive SARS-CoV-2 test to the end of the follow-up. Results from one SARS-CoV-2 infection (*n* = 234,990), two SARS-CoV-2 infections (*n* = 28,509) and three or more SARS-CoV-2 infections (*n* = 1,023) versus noninfected controls (*n* = 5,334,729), in those with a first infection before the Omicron wave, are compared. Adjusted HRs (dots) and 95% CIs (error bars) are presented, as are the estimated excess burden (bars) and 95% CIs (error bars). Burdens are presented per 1,000 persons at 1 year of follow-up.

To better understand the cumulative risks incurred by people with multiple infections, we estimated the cumulative risk and burden of a set of prespecified outcomes in those who did not have a reinfection (had only one infection), and those who had two or three or more infections during the 1-year period after the acute phase of the first infection, compared to a noninfected control group. Cohort characteristics are provided in Supplementary Table 6. There was a graded association in that the risks of adverse health outcomes increased as the number of infections increased. Compared to the noninfected control group, those who only had one infection had an increased risk of at least one sequela (HR = 1.37, 95% CI = 1.36–1.38; burden per 1,000 persons at one-year = 108.88, 95% CI = 105.89–111.87); the risk was higher in those who had two infections (HR = 2.07, 95% CI = 2.03–2.11; burden = 260.41, 95% CI = 253.70–267.09) and highest in those with three or more infections (HR = 2.35, 95% CI = 2.12–2.62; burden = 305.44, 95% CI = 268.07–341.11). In a pairwise comparison of those with two infections versus one infection, those with two infections had an increased risk of at least one sequela (HR = 1.51, 95% CI = 1.48–1.54; burden = 151.53, 95% CI = 144.83–158.21); in pairwise comparison of those with three or more infections versus those with only two infections, those with three or more infections had a higher risk of at least one sequela (HR = 1.14, 95% CI = 1.02–1.27; burden = 45.02, 95% CI = 7.66–80.70). Results were consistent when hospitalization and sequelae by organ system were examined (Fig. 5 and Supplementary Tables 7–12).

### Positive and negative outcome controls

We conducted a positive outcome control analysis to examine whether our approach reproduced previous established knowledge, testing whether the association of a SARS-CoV-2 infection (irrespective of reinfection) was associated with risk of fatigue (a well-characterized, cardinal postacute sequela of COVID-19, where a positive association would be expected based on previous evidence). Results showed that, compared to a noninfected control group, those with a SARS-CoV-2 infection exhibited an increased risk of fatigue (HR = 1.72, 95% CI = 1.70–1.74).

We then conducted a set of negative outcome control analyses to test for the potential presence of spurious associations using the same data sources, cohort construction processes, covariate selections and definitions (including predefined and algorithmically selected high-dimensional covariates), covariate balance methods and result interpretations as those of our primary analysis. Results examining the risk of atopic dermatitis and neoplasms (negative outcome controls), where there was no previous biological or epidemiological evidence to suggest an association should be expected, did not show a significant association in those who had reinfection compared to those with no reinfection (HR = 1.06, 95% CI = 0.91–1.24 and HR = 1.03, 95% CI = 0.97–1.10, respectively).

## Discussion

In this study of 5,819,264 people, including 443,588 people with a first infection, 40,947 people who had reinfection and 5,334,729 noninfected controls, we showed that compared to people with no reinfection, people who had reinfection exhibited increased risks of all-cause mortality, hospitalization and several prespecified outcomes. The risks were evident in those who were unvaccinated and had one vaccination or two or more vaccinations before reinfection. The risks were most pronounced in the acute phase but persisted in the postacute phase of reinfection, and risks for all sequelae were still evident at 6 months. Compared to noninfected controls, assessment of the cumulative risks of repeat infection showed that the risk and burden of all-cause mortality and the prespecified health outcomes increased in a graded fashion according to the number of infections (that is, risks were lowest in people with one infection, increased in people with two infections and were highest in people with three or more infections). Altogether, the findings show that reinfection further increases risks of all-cause mortality and adverse health outcomes in both the acute and postacute phases of reinfection. The findings highlight the clinical consequences of reinfection and emphasize the importance of preventing reinfection by SARS-CoV-2.

Estimates suggest that more than half a billion people around the globe have been infected with SARS-CoV-2 at least once^11^. For the large and growing number of people who encountered a first infection, the question of whether a second infection carries additional risks is important. In this work, we showed that reinfection further increases risks of all-cause mortality and adverse health outcomes in both the acute and postacute phases of reinfection, suggesting that for people who have already been infected once, continued vigilance to reduce the risk of reinfection may be important to lessen the overall risk to one’s health.

Given the likelihood that SARS-CoV-2 will continue to mutate and might remain a threat for years if not decades, leading to the emergence of variants or subvariants that might be more immune-evasive, and given that reinfections are occurring and might continue to occur due to these emerging SARS-CoV-2 variants at scale in many countries across the globe, and given that reinfection contributes nontrivial health risk both in the acute and postacute phases, a strategy that would result in vaccines that are more durable, cover a broad array of variants (variant-proof vaccine strategy), reduce transmission (and subsequently reduce the risk of infection and reinfection) and reduce both acute and long-term consequences in people who get infected or reinfected is urgently needed^12^. Other pharmaceutical and nonpharmaceutical interventions to lessen both the risk of reinfection and its adverse health consequences are also urgently needed.

Questions have been raised with regard to whether reinfection increases the risk of long COVID—the umbrella term encompassing the postacute sequelae of SARS-CoV-2 infection. Our results show that beyond the acute phase, reinfection with SARS-CoV-2 contributes substantial additional risks of all-cause mortality, hospitalization and postacute sequelae in the pulmonary and broad array of extra pulmonary organ systems.

The mechanisms underpinning the increased risks of death and adverse health outcomes in reinfection are not completely clear. Previous exposure to the virus may be expected to hypothetically reduce risk of reinfection and its severity^9,13^; however, SARS-CoV-2 is mutating rapidly and new variants and subvariants are replacing older ones every few months. Evidence suggests that the reinfection risk is especially higher with the Omicron variant, which was shown to have a marked ability to evade immunity from previous infection^10,14^. Any protection from previous infection (against reinfection and its severity) also wanes over time^10^; evidence suggests that protection from reinfection declined as time increased since the last immunity-conferring event in people who had previously been infected with SARS-CoV-2, regardless of vaccination status^15^. Furthermore, impaired health as a consequence of the first infection might result in increased risk of adverse health consequences upon reinfection. Our results expand this evidence base and show that in people who get reinfected, reinfection (compared to no reinfection) further increases risk in both the acute and postacute phases and that this was evident even among fully vaccinated people, suggesting that even combined (a hybrid of) natural immunity (from previous infection) and vaccine-induced immunity does not abrogate the risk of adverse health effects after reinfection. The totality of evidence suggests that strategies to prevent reinfection might benefit people regardless of previous history of infection and vaccination status.

This study has several strengths. To our knowledge, this is the first study to characterize both the short- and long-term health risks of reinfection. We used the US Department of Veterans Affairs national healthcare database (the largest nationally integrated healthcare delivery system in the US) to undertake the analyses. We used advanced statistical methodologies and adjusted through weighting for a set of predefined covariates selected based on previous knowledge and algorithmically selected covariates from high-dimensional data domains including diagnoses, prescription records and laboratory test results. Because the virus is mutating over time and the proportion of different variants may vary geographically, and because different variants may have different effects on outcomes, we further adjusted our analyses for measures of the time and geographical region where participants first tested positive for SARS-Cov-2 and additionally for the proportions of each variant at the time and region of their first infection. We evaluated both acute and postacute outcomes of reinfection and examined risks according to vaccination status before reinfection. We evaluated the rigor of our approach by testing positive and negative outcome controls to determine whether our approach would produce results consistent with pretest expectations.

The study has several limitations. The cohorts of people with one, two, three or more infections included those that had a positive test for SARS-CoV-2 and did not include those who may have had an infection with SARS-CoV-2 but were not tested; this may have resulted in misclassification of exposure since these people would have been enrolled in the control groups. If present in large numbers and if their true risk of adverse health outcomes is substantially higher than the noninfected controls, then this may have resulted in underestimation of the risks of reinfection. Although we leveraged several Veterans Affairs and non-Veterans Affairs data sources, our datasets may not have comprehensively captured care received outside the Veterans Affairs (including exposure (positive SARS-CoV-2), covariates (for example, vaccination) and outcomes), which may contribute to potential misclassification. Although the Veterans Affairs population which consists of those who are mostly older and male may not be representative of the general population, our cohorts included 10.3% women, which amounted to 589,573 participants, and 12% were under 38.8 years of age (the median age of the US population in 2021), which amounted to 680,358 participants. Subgroup analyses were not conducted by age, sex and race. Although we balanced characteristics of the exposure groups through weighting using a set of predefined and algorithmically selected covariates, which included demographic, behavioral, contextual and clinical characteristics, we cannot completely rule out residual confounding from unmeasured or otherwise unknown confounders. The COVID-19 pandemic is a highly dynamic global event that is still unfolding in real time; as various epidemiological drivers of this pandemic change over time (including emergence of new variants, increase in vaccine uptake and waning vaccine immunity), it is likely that the epidemiology of reinfection and its health consequences may also change over time. The aim of our analyses was to examine the health risks associated with those individuals who had reinfection (compared to no reinfection). Our analyses should not be interpreted as an assessment of severity of a second infection versus that of a first infection, nor should they be interpreted as an examination of the risks of adverse health outcomes after a second infection compared to risks incurred after a first infection. Our analyses do not provide a comparative assessment of the risks of reinfection with different variants or subvariants.

In sum, in this study of 5,819,264 individuals, we provide evidence that reinfection contributes to additional health risks beyond those incurred in the first infection including all-cause mortality, hospitalization and sequelae in a broad array of organ systems. The risks were evident in the acute and postacute phases of reinfection. The evidence suggests that for people who already had a first infection, prevention of a second infection may protect from additional health risks. Prevention of infection and reinfection with SARS-CoV-2 should continue to be the goal of public health policy.

## Methods

### Ethics statement

This study was approved by the institutional review board of the Veterans Affairs St. Louis Health Care System, which granted a waiver of informed consent (protocol no. 1606333). All participants who were eligible for this study were enrolled; no a priori sample size analyses were conducted to guide enrollment. All analyses were observational, and investigators were aware of participant exposure and outcome status.

### Setting

Participants were selected from the US Veterans Health Administration (VHA) electronic health database. The VHA delivers healthcare to discharged Veterans of the US armed forces in a network of nationally integrated healthcare systems including more than 1,415 healthcare facilities. Veterans enrolled for care in the VHA have access to extensive medical benefits, such as inpatient and outpatient services, preventative, primary and specialty care, mental health services, geriatric care, long-term and home healthcare, medications and medical equation and prosthetics. The VHA electronic health database is updated daily.

### Cohorts

A flowchart of cohort construction is provided in Extended Data Fig. 1. We first identified users of the VHA with at least one positive SARS-CoV-2 test between 1 March 2020 and 6 April 2022 (*n* = 519,767), enrolling these participants at the date of first positive test (set as *T*_0_). Use of the VHA was defined as having record of use of outpatient or inpatient service, receipt of medication or use of laboratory service with the VHA healthcare system in the 2 years before enrollment. We selected those still alive 90 d after their first positive SARS-CoV-2 test (*n* = 489,779). We then further selected participants who experienced reinfection, defined as a positive SARS-CoV-2 test 90 d or more after the first infection, where reinfection could occur between 1 June 2020 and 25 June 2022, which spans the time frame in the US in which pre-Delta, Delta and Omicron variants predominated^16–19^. The 90-d minimum time frame to define reinfection was specified to minimize inclusion of repeat positive tests that may be related to the first infection^16–19^. There were 40,947 participants who had a reinfection, where the time of reinfection was set as *T*_1_. To ensure a similar distribution of follow-up time in the no reinfection and reinfection groups, participants in the no reinfection group were randomly assigned a *T*_1_ based on the distribution of *T*_1_ of those in the reinfection group who shared the same calendar month as the date of first infection, resulting in a group of 443,588 participants with no reinfection that were alive at their assigned *T*_1_.

We then constructed a noninfected control group. We first identified 5,760,792 VHA users between 1 March 2020 and 6 April 2022 with no record of a positive SARS-CoV-2 test. We then randomly assigned a *T*_0_ to each participant in the group on the basis of the distribution of the *T*_0_ dates in those with at least one positive SARS-CoV-2 test, selecting the 5,458,815 who were alive at their assigned *T*_0_. We selected those who were alive 90 d after their *T*_0_ (*n* = 5,408,880). After randomly assigning a *T*_1_, there were 5,334,729 in the noninfected control cohort. All cohort participants were followed until 25 June 2022.

### Data sources

Participant data were obtained from the VHA Corporate Data Warehouse. The patient and vital status domains provided data on demographic characteristics. VHA mortality information contains both inhospital and nonhospital deaths collected from the Veterans Affairs and non-Veterans Affairs sources including the VHA’s Beneficiary Identification Record Locator System and medical inpatient datasets, as well as Medicare Vital Status File, Social Security Administration’s Master File and information from death certificates and the National Cemetery Administration^20^. The outpatient and inpatient encounter domains provided information on health characteristics including details on date and place of encounter with the healthcare system and diagnostic and procedural information. The Pharmacy and Bar Code Medication Administration domains provided medication records, while the laboratory results domain provided laboratory test results for tests conducted in both inpatient and outpatient settings^7,21^. Information about SARS-CoV-2 tests and vaccinations were obtained from the COVID-19 Shared Data Resource. Positive SARS-CoV-2 tests consisted of results from PCR or antigen tests conducted in the Veterans Affairs or reported to the Veterans Affairs. The 2019 Area Deprivation Index at the residential address of each cohort participant was used as a contextual measure of socioeconomic disadvantage^22^. Information from the US Center for Disease Control and Prevention provided the proportion of SARS-CoV-2 variant by week in each Health and Human Services (HHS) region.

### Outcomes

Outcomes were prespecified on the basis of previous evidence^1–8,21,23–29^. Outcomes included all-cause mortality, hospitalization, having at least one sequela and organ system disorders including cardiovascular disorders, coagulation and hematological disorders, diabetes, fatigue, gastrointestinal disorders, kidney disorders, mental health disorders, musculoskeletal disorders, neurological disorders and pulmonary disorders. Organ system disorders were defined as a composite outcome of a set of prespecified individual sequelae in that system at the date of first incident sequela in that system during follow-up. Organ system disorders were defined on the basis of inpatient or outpatient diagnostic codes, medication prescriptions or laboratory values. A list of the individual sequelae by organ system are provided in Supplementary Table 13. The outcome of ‘at least one sequela’ was defined at the time of occurrence of first incident sequela among all individual sequelae. For a participant, for a given outcome, each individual sequela was included in the assessed outcome only when there was no record of that health condition in the 2 years before *T*_0_. Participants were excluded from the analysis of an outcome if they had previous history of all the individual sequelae that contributed to the outcome being examined. Hospitalization was defined as first inpatient admittance during follow-up. In analyses of kidney disorders, participants with a previous history of end-stage kidney disease were excluded and follow-up was censored at the time of end-stage kidney disease (Supplementary Table 13).

### Covariates

Covariates included a set of variables that were predefined based on previous knowledge^4–7,21,23,25–27,30–33^ and a set of variables that were selected algorithmically. Predefined covariates included demographic information (age, race and sex), contextual information (Area Deprivation Index) and measures of healthcare use in the 2 years before *T*_0_, which included the number of outpatient visits, inpatient visits, unique medication prescriptions, routine laboratory blood panels and use of Medicare services, as well as a previous history of receiving an influenza vaccination. Smoking status was also included as a covariate. Characteristics of the participants’ health history included record of anxiety, cancer, cardiovascular disease, cerebrovascular disease, chronic kidney disease, chronic obstructive pulmonary disease, dementia, depression, type 2 diabetes mellitus, estimated glomerular filtration rate, immunocompromised status, peripheral artery disease, systolic and diastolic blood pressure and body mass index on the basis of inpatient or outpatient diagnostic codes, medication prescriptions, laboratory values and vital signs. Immunocompromised status was defined according to the US Center for Disease Control and Prevention definitions by a history of organ transplantation, advanced kidney disease (an estimated glomerular filtration rate <15 ml min ^−1^1.73 m^−^^2^ or end-stage renal disease), cancer, HIV or conditions with prescriptions of more than 30-d use of corticosteroids or immunosuppressants including systemic lupus erythematosus and rheumatoid arthritis.

We also included a set of covariates related to the acute phase of the first infection: severity of the acute phase of the disease, defined in mutually exclusive groups of nonhospitalized, hospitalized and admitted to the intensive care unit during the acute phase and whether the participant received SARS-CoV-2 treatment of antivirals, antibodies and immunomodulators including corticosteroids, interleukin-6 inhibitors and kinase inhibitors. We also included—as measures of spatiotemporal differences—the calendar week of enrollment and geographical region of receipt of care defined by the Veterans Integrated Services Networks. We also adjusted for vaccination status, which was defined as receiving no, one, two and three or more Janssen (Ad26.COV2.S; Johnson & Johnson), Pfizer-BioNTech (BNT162b2) or Moderna (mRNA-1273) vaccination shots. In consideration of the dynamicity of the pandemic, additional covariates included hospital system capacity (the total number of inpatient hospital beds), inpatient bed occupancy rates (the percentage of hospital beds that were occupied) and a measure of the proportions of SARS-CoV-2 variants by HHS region^33^. These measures were ascertained for each participant in the week of cohort enrollment at the location of the healthcare system they received care at.

In addition to the predefined covariates, we leveraged the high dimensionality of Veterans Affairs electronic health records by employing a high-dimensional variable selection algorithm to identify additional covariates that may potentially confound the examined associations^34^. We used the diagnostic classifications system from the Clinical Classifications Software Refined v.2021.1, available from the Healthcare Cost and Utilization Project sponsored by the Agency for Healthcare Research and Quality, to classify more than 70,000 International Classification of Diseases, 10th revision diagnosis codes in the 2 years before *T*_0_ for each participant into 540 diagnostic categories^35–37^. Using the Veterans Affairs national drug classification system, we also classified 3,425 different medications into 543 medication classes^38,39^. Finally, on the basis of Logical Observation Identifiers Names and Codes, we classified laboratory results from 38 different laboratory measurements into 62 laboratory test abnormalities, defined by being above or below the corresponding reference ranges. Of the high-dimensional variables that occurred at least 100 times in participants in each group, we selected the top 100 variables with the highest relative risk for differences in group membership in first infection or reinfection.

### Statistical analysis

Mean (s.d.) and frequency (percentage) of characteristics are reported for those with no SARS-CoV-2 reinfection, SARS-CoV-2 reinfection and the noninfected control group, where appropriate. We provide information on the distribution of frequency of reinfections, time between infections and variant of reinfection (defined by predominant variant given the calendar week and HHS region of the residential location of cohort participants when reinfection occurred).

All associations were estimated based on weighting approaches combined with survival analyses. We conducted a primary analysis to evaluate the risk and burden of reinfection compared to no reinfection (Supplementary Fig. 2). Logistic regressions were constructed to estimate the propensity score of group membership; regressions included predefined covariates, high-dimensional covariates and time from *T*_0_ to *T*_1_ as a means to adjust for residual differences in duration of follow-up. A reference cohort of the overall infected cohort at *T*_0_ was used as the target population. Inverse probability weighting was then used to balance the covariates. A weighted Cox survival model was then used to estimate the average risk and event rate difference between those with a reinfection and those with no reinfection. Standard errors were estimated by applying the robust sandwich variance estimator method. Covariate balance among all predefined and high-dimensional variables were assessed through the standardized mean difference, where a difference <0.1 was taken as evidence of balance. We estimated the incidence rate difference (referred to as excess burden) between groups per 1,000 participants at 6 months after the start of the follow-up based on the difference in survival probability between the groups. These analyses were repeated by subgroup on the basis of the number of vaccination shots received (0, 1 or 2+) before reinfection using an overlap weighting approach. To test whether the risk on the multiplicative scale differed between participants with different duration between *T*_0_ and *T*_1_, a model with a linear interaction term between reinfection status and duration was constructed and the corresponding *P* value is reported for the outcomes of all-cause mortality, hospitalization and having at least one sequela.

To examine whether risks associated with a reinfection were present in the acute and postacute phases of reinfection, we conducted analyses to examine risks in 30-d time intervals starting at the time of reinfection up to 180 d after reinfection. HRs and 30-d burdens were estimated independently for each 30-d time interval. During each 30-d interval, outcomes were defined at the time of first occurrence within this interval in those who did not have that outcome in the 2 years before the first infection.

We then used a doubly robust approach to examine the risk and cumulative burden per 1,000 persons at one-year after first infection of sequelae associated with one, two and three or more infections versus a noninfected control (Supplementary Fig. 3). A third or more infection was defined as a positive test at least 90 d after the second infection. The number of infections and outcomes were assessed in the 360 d after *T*_0_ + 30 d. Since those with three or more infections predominantly had their first infection before the Omicron variant was present, to enhance comparison across groups, we restricted this analysis to those with a *T*_0_ period before Omicron became the predominant variant in at least one HHS region (11 December 2022). Because participants with three or more infections must have not died during the follow-up period to have that third (or more) infection, we did not examine the outcome of all-cause mortality due to immortal time bias.

### Positive and negative controls

We examined, as positive outcome controls, the risk of fatigue in those with a SARS-CoV-2 infection compared to the noninfected control group as a means of testing whether our approach would reproduce established knowledge^4,5,25–27^.

The application of a negative outcome control may help detect both suspected and unsuspected sources of spurious biases. Therefore, we examined the difference in risks of atopic dermatitis and neoplasms between those who had reinfection and the first infection, where no previous knowledge suggested that an association should be expected. The testing of positive and negative outcome controls may lessen, although not eliminate, concerns about biases related to study design, covariate selection, analytical approach, outcome ascertainment, unmeasured confounding and other potential sources of latent biases^40,41^.

All analyses were two-sided. In all analyses, a 95% CI that excluded unity was considered evidence of statistical significance. All analyses were conducted in SAS Enterprise Guide v.8.2, and all figures were generated in R v.4.0.4.

### Reporting summary

Further information on research design is available in the Nature Research Reporting Summary linked to this article.

## Online content

Any methods, additional references, Nature Research reporting summaries, source data, extended data, supplementary information, acknowledgements, peer review information; details of author contributions and competing interests; and statements of data and code availability are available at 10.1038/s41591-022-02051-3.

## Supplementary information

Supplementary Information Supplementary TOC and Figs. 1–3.

Reporting Summary

Supplementary Data 1 Supplementary Data Tables 1–13.

## Data Availability

The data that support the findings of this study are available from the US Department of Veterans Affairs. Veterans Affairs data are made freely available to researchers behind the Veterans Affairs firewall with an approved Veterans Affairs study protocol. For more information, please visit https://www.virec.research.va.gov or contact the Veterans Affairs Information Resource Center at VIReC@va.gov.

## Code availability

The code used for the analysis is available at https://github.com/BcBowe3.
