# Supplementary material for: Acute and postacute sequelae associated with SARS-CoV-2 reinfection
Source: Nat Med. 2022 Nov 10;28(11):2398–405. doi: 10.1038/s41591-022-02051-3 (PMC9671810; doi:10.1038/s41591-022-02051-3)

nature medicine

Article

https://doi.org/10.1038/s41591-022-02051-3

Acute and postacute sequelae associated with SARS-CoV-2 reinfection

In the format provided by the authors and unedited

Supplementary Information

Outcomes of SARS-CoV-2 Reinfection

Table of Contents	Page
**Supplementary Figures**	
**Supplementary Figure 1:** Standardized mean differences between SARS-CoV-2 reinfection and no reinfection before and after weighting.	2
**Supplementary Figure 2:** Timeline of analyses for risk and burden assessment comparing reinfection to no reinfection.	3
**Supplementary Figure 3:** Timeline of analyses for risk and burden assessment by number of infections.	4
**Supplementary Tables**	
**Supplementary Table 1:** Demographic and health characteristics of those with non-infected control group, no SARS-CoV-2 reinfection, and SARS-CoV-2 reinfection.	Excel sheet
**Supplementary Table 2:** Demographic and health characteristics by SARS-CoV-2 reinfection status after weighting.	Excel sheet
**Supplementary Table 3** Risk and 6-month burden of death, hospitalization, at least one sequela, and sequelae by organ system in SARS-CoV-2 reinfection compared to those with no SARS-CoV-2 reinfection.	Excel sheet
**Supplementary Table 4:** Risk and 6-month burden of death, hospitalization, at least one sequela, and sequelae by organ system in SARS-CoV-2 reinfection compared to no SARS-CoV-2 reinfection by vaccination status at time of reinfection.	Excel sheet
**Supplementary Table 5:** Risk and 6-month burden of death, hospitalization, at least one sequela, and sequelae by organ system in SARS-CoV-2 reinfection compared to no SARS-CoV-2 reinfection by time since reinfection.	Excel sheet
**Supplementary Table 6:** Demographic and health characteristics in those with one infection, two infections, three infections, and the non-infected control.	Excel sheet
**Supplementary Table 7** Risk and one year burden of hospitalization, at least one sequela, and sequelae by organ system in those with one SARS-CoV-2 infection compared to the non-infected control.	Excel sheet
**Supplementary Table 8** Risk and one year burden of hospitalization, at least one sequela, and sequelae by organ system in those with two SARS-CoV-2 infections compared to the non-infected control.	Excel sheet
**Supplementary Table 9** Risk and one year burden of hospitalization, at least one sequela, and sequelae by organ system in those with three or more SARS-CoV-2 infections compared to the non-infected control.	Excel sheet
**Supplementary Table 10** Risk and one year burden of hospitalization, at least one sequela, and sequelae by organ system in those with two SARS-CoV-2 infections compared to those with one SARS-CoV-2 infection.	Excel sheet
**Supplementary Table 11** Risk and one year burden of hospitalization, at least one sequela, and sequelae by organ system in those with three or more SARS-CoV-2 infections compared to those with one SARS-CoV-2 infection.	Excel sheet

**Supplementary Table 12**<br>Risk and one year burden of hospitalization, at least one sequela, and sequelae by organ system in those with three or more SARS-CoV-2 infections compared to those with two SARS-CoV-2 infections.	Excel sheet
**Supplementary Table 13**<br>Organ system disorder definitions	Excel sheet

Supplementary Figure 1: Standardized mean differences between SARS-CoV-2 reinfection and those without reinfection (only one infection) before and after weighting.

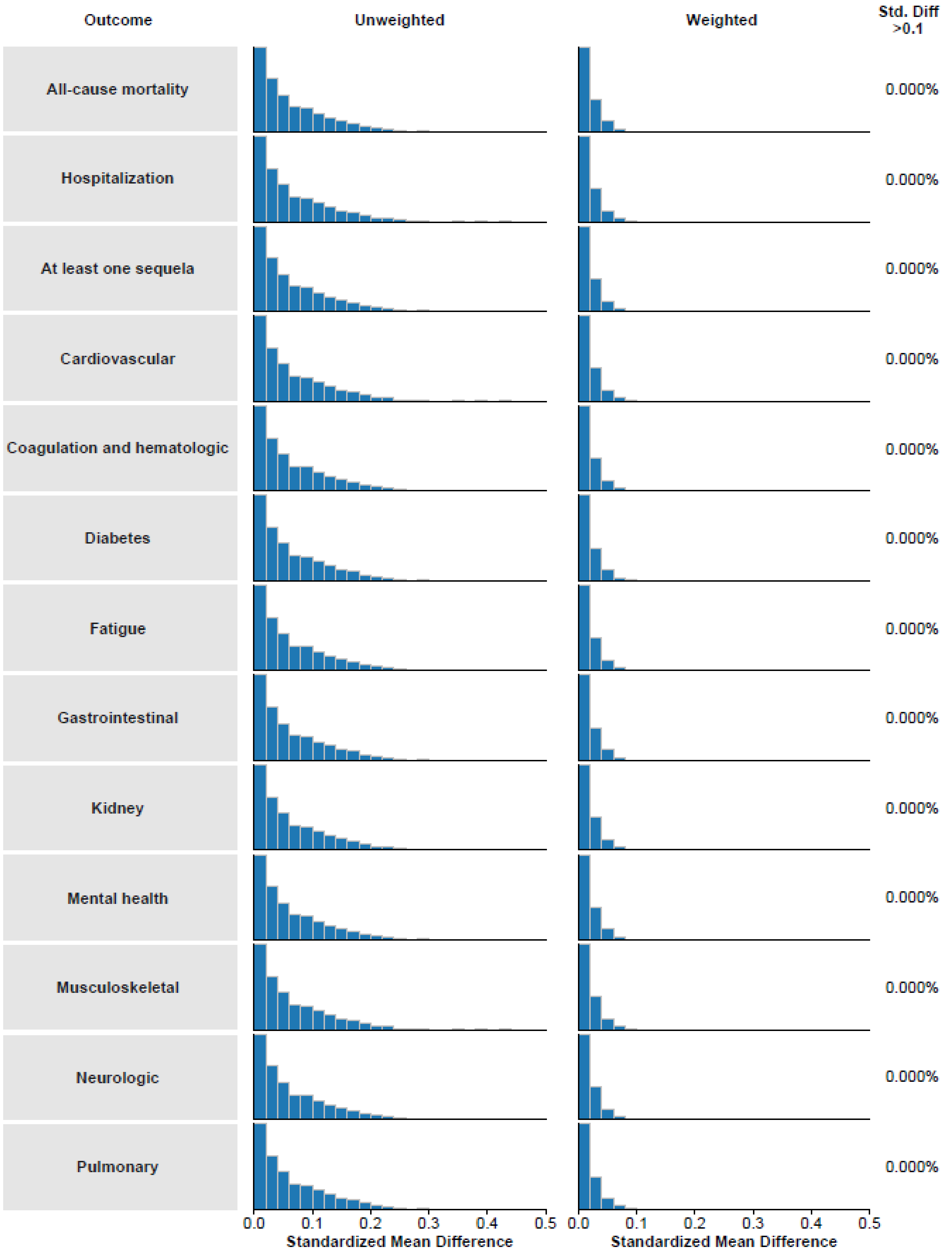

Supplementary Figure 2: Timeline of analyses for risk and burden assessment comparing reinfection to first infection. Timelines are plotted for the first infection and reinfection cohorts. Cohort participant characteristics were balanced at T_0, the date of their first infection. Risk and burdens were then estimated in the 180 days following T_1, the date of reinfection, in comparison to a similar T_1 in the no reinfection group.

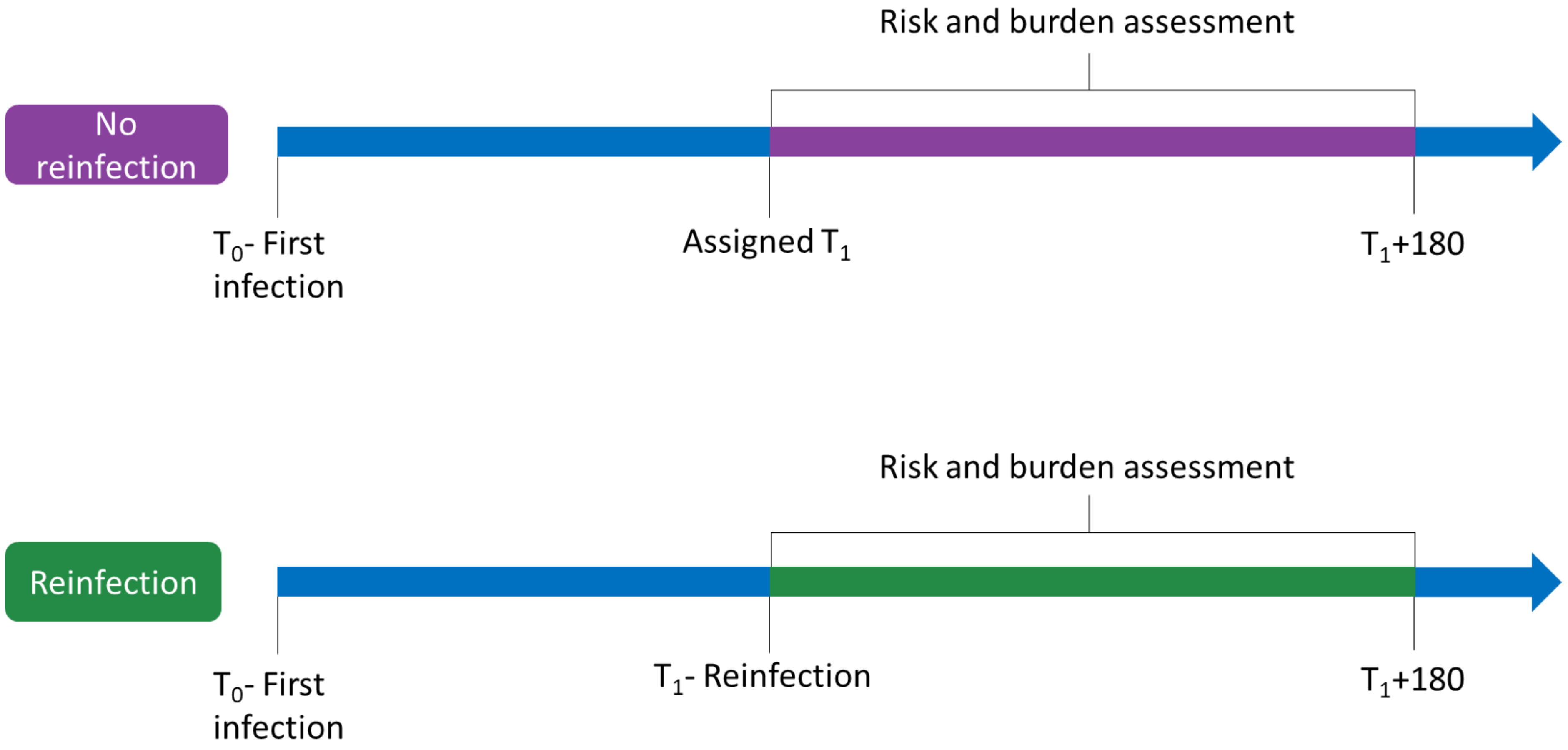

Supplementary Figure 3: Timeline of analyses for risk and burden assessment by number of infections. Timelines are plotted for the non-infected control, and in those with one, two, and three or more infections. Cohort participant characteristics were balanced at T_0, the date of their first infection or a randomly assigned T_0 in the non-infected control group. Risk and burdens were then estimated in the 360 days following the acute phase of the first infection (or corresponding time in the non-infected control group).

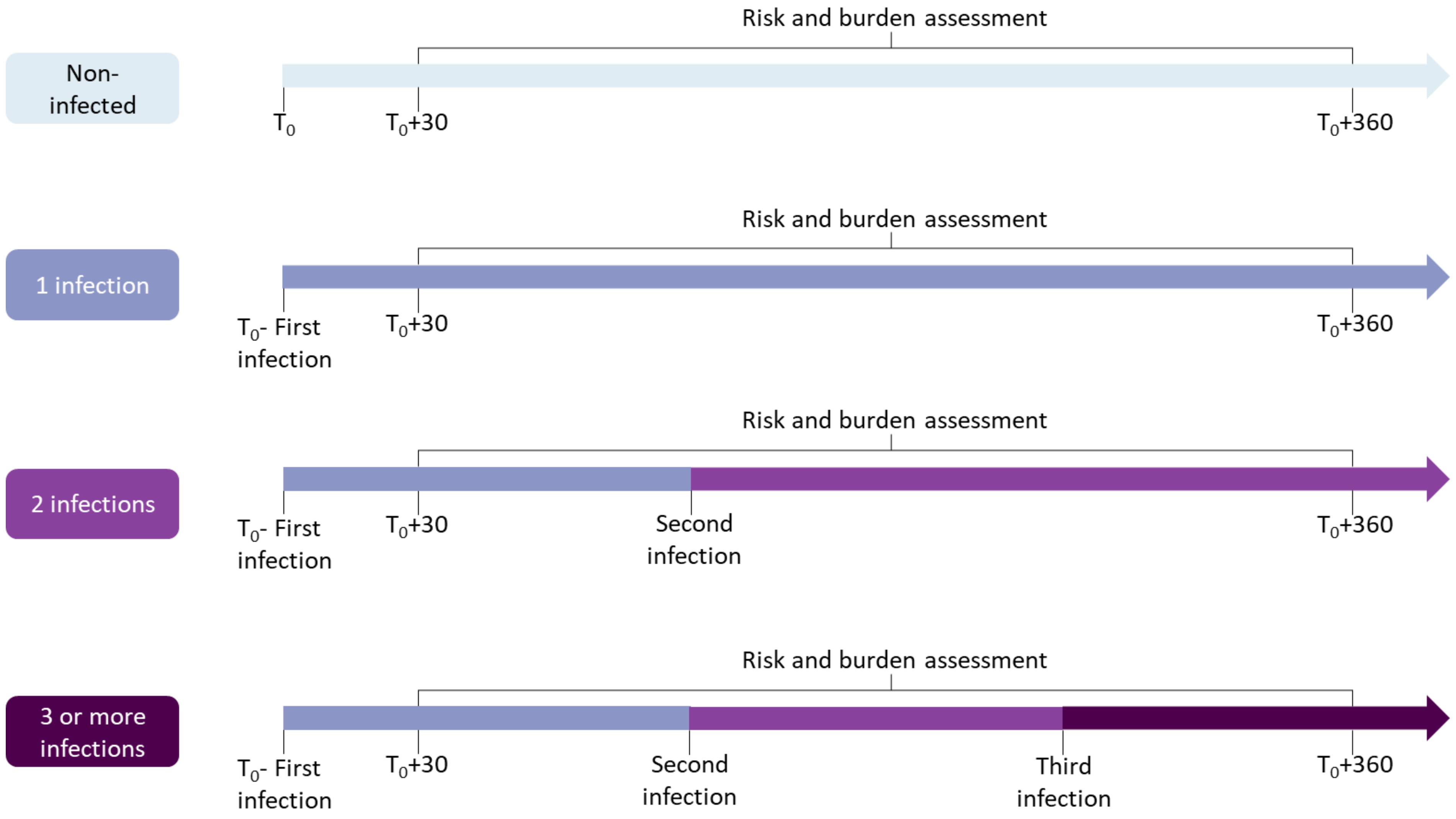

Supplement: Supplementary file 1 — Supplementary TOC and Figs. 1–3. [file 41591_2022_2051_MOESM1_ESM.pdf]
